# Dynamic subcellular localization of the mono-ADP-ribosyltransferase ARTD10 and interaction with the ubiquitin receptor p62

**DOI:** 10.1186/1478-811X-10-28

**Published:** 2012-09-20

**Authors:** Henning Kleine, Andreas Herrmann, Trond Lamark, Alexandra H Forst, Patricia Verheugd, Juliane Lüscher-Firzlaff, Barbara Lippok, Karla LH Feijs, Nicolas Herzog, Elisabeth Kremmer, Terje Johansen, Gerhard Müller-Newen, Bernhard Lüscher

**Affiliations:** 1 Institute of Biochemistry and Molecular Biology, Medical School, RWTH Aachen University, Pauwelsstraße 30, Aachen, 52074, Germany; 2 Molecular Cancer Research Group, Institute of Medical Biology, University of Tromsø, Tromsø, 9037, Norway; 3 Helmholtz Zentrum München, Institut für Molekulare Immunologie, Marchioninistr. 25, München, 81377, Germany; 4 Present address: Abbott GmbH & Co. KG, Max-Planck-Ring 2a, Wiesbaden, 65205, Germany; 5 Present address: Beckman Research Institute, City of Hope National Medical Center, Duarte, CA, 91010, USA

**Keywords:** ARTD10/PARP10, Autophagy, FRAP, iFLAP, Live-cell imaging, NES, NLS, Nucleocytoplasmic shuttling, SQSTM1

## Abstract

**Background:**

ADP-ribosylation is a posttranslational modification catalyzed in cells by ADP-ribosyltransferases (ARTD or PARP enzymes). The ARTD family consists of 17 members. Some ARTDs modify their substrates by adding ADP-ribose in an iterative process, thereby synthesizing ADP-ribose polymers, the best-studied example being ARTD1/PARP1. Other ARTDs appear to mono-ADP-ribosylate their substrates and are unable to form polymers. The founding member of this latter subclass is ARTD10/PARP10, which we identified as an interaction partner of the nuclear oncoprotein MYC. Biochemically ARTD10 uses substrate-assisted catalysis to modify its substrates. Our previous studies indicated that ARTD10 may shuttle between the nuclear and cytoplasmic compartments. We have now addressed this in more detail.

**Results:**

We have characterized the subcellular localization of ARTD10 using live-cell imaging techniques. ARTD10 shuttles between the cytoplasmic and nuclear compartments. When nuclear, ARTD10 can interact with MYC as measured by bimolecular fluorescence complementation. The shuttling is controlled by a Crm1-dependent nuclear export sequence and a central ARTD10 region that promotes nuclear localization. The latter lacks a classical nuclear localization sequence and does not promote full nuclear localization. Rather this non-conventional nuclear localization sequence results in an equal distribution of ARTD10 between the cytoplasmic and the nuclear compartments. ARTD10 forms discrete and dynamic bodies primarily in the cytoplasm but also in the nucleus. These contain poly-ubiquitin and co-localize in part with structures containing the poly-ubiquitin receptor p62/SQSTM1. The co-localization depends on the ubiquitin-associated domain of p62, which mediates interaction with poly-ubiquitin.

**Conclusions:**

Our findings demonstrate that ARTD10 is a highly dynamic protein. It shuttles between the nuclear and cytosolic compartments dependent on a classical nuclear export sequence and a domain that mediates nuclear uptake. Moreover ARTD10 forms discrete bodies that exchange subunits rapidly. These bodies associate at least in part with the poly-ubiquitin receptor p62. Because this protein is involved in the uptake of cargo into autophagosomes, our results suggest a link between the formation of ARTD10 bodies and autophagy.

**Lay abstract:**

Post-translational modifications refer to changes in the chemical appearance of proteins and occur, as the name implies, after proteins have been synthesized. These modifications frequently affect the behavior of proteins, including alterations in their activity or their subcellular localization. One of these modifications is the addition of ADP-ribose to a substrate from the cofactor NAD^+^. The enzymes responsible for this reaction are ADP-ribosyltransferases (ARTDs or previously named PARPs). Presently we know very little about the role of mono-ADP-ribosylation of proteins that occurs in cells. We identified ARTD10, a mono-ADP-ribosyltransferase, as an interaction partner of the oncoprotein MYC. In this study we have analyzed how ARTD10 moves within a cell. By using different live-cell imaging technologies that allow us to follow the position of ARTD10 molecules over time, we found that ARTD10 shuttles constantly in and out of the nucleus. In the cytosol ARTD10 forms distinct structures or bodies that themselves are moving within the cell and that exchange ARTD10 subunits rapidly. We have identified the regions within ARTD10 that are required for these movements. Moreover we defined these bodies as structures that interact with p62. This protein is known to play a role in bringing other proteins to a structure referred to as the autophagosome, which is involved in eliminating debris in cells. Thus our work suggests that ARTD10 might be involved in and is regulated by ADP-riboslyation autophagosomal processes.

## Background

ARTD10 (formerly PARP10) is an intracellular mono-ADP-ribosyltransferase that was identified as an interaction partner of the oncoprotein MYC [1]. ADP-ribosylation is a posttranslational modification that controls both intra- and extracellular processes [2]. Intracellular ADP-ribosylation involves a family of 17 ADP-ribosyltransferases (ARTDs) that can be divided into three classes. The six enzymes of the first have the capacity to synthesize ADP-ribose polymers, with ARTD1/PARP1 being the best studied family member [3]. The nine ARTDs in class 2 lack the catalytic glutamate residue in the active center found in class 1. This glutamate appears to be critical for the ability to form ADP-ribose polymers. Thus the class 2 enzymes are thought to mono-ADP-ribosylate their substrates but are unable to synthesize ADP-ribose polymers, with ARTD10 being the defining enzyme of this class [4]. Finally, the two class 3 proteins are most likely inactive due to alterations in the catalytic center that prevent binding of NAD^+^. The analysis of recognizable domains within the ARTD family suggests that these proteins participate in many different physiological processes [2], implying that ADP-ribosylation is a widespread posttranslational modification.

Indeed poly-ADP-ribosylation has been recognized to regulate different processes, including DNA repair, transcription, genome stability, and signaling. For example ARTD1, which is predominantly nuclear, is involved in the regulation of DNA repair [3]. The enzymatic activity of ARTD1 is stimulated upon binding to damaged DNA, which results in the local generation of ADP-ribose polymers. These serve as binding sites for DNA repair proteins that possess specific poly-ADP-ribose recognition domains [5-9]. Moreover poly-ADP-ribose can be detached from ARTD1, migrate into the cytoplasm, and stimulate the release of the death effector apoptosis-inducing factor (AIF) from mitochondria, thereby promoting apoptosis [10]. Other examples are ARTD5 and 6 (or tankyrase 1 and 2), enzymes that were initially identified to control telomere length and sister chromatin separation [11]. More recent findings revealed that ARTD5/6 also regulate WNT signaling by poly-ADP-ribosylating axin, which serves as a key scaffold protein to control the function of β-catenin in the cytoplasm. Poly-ADP-ribosylation of axin promotes binding of the E3 ligase RNF146, subsequently resulting in the degradation of axin by the ubiquitin-proteasome system and the activation of β-catenin [12,13]. Similarly to axin, the stability of 3BP2 is also controlled by ARTD5/6-dependent poly-ADP-ribosylation and recruitment of RNF146 [14]. Together these findings suggest that modification by ADP-ribose polymers along with the responsible enzymes can be found in both the nucleus and the cytoplasm.

It is less clear, however, where mono-ADP-ribosyltransferases are located and thus where mono-ADP-ribosylation occurs in cells. The identification of ARTD10 as an interaction partner of the oncoprotein MYC suggested that this enzyme is localized in the nuclear compartment. However our initial findings revealed that ARTD10 is primarily cytoplasmic under steady-state conditions, controlled at least in part by a nuclear export sequence (NES) [1]. We have now further analyzed the subcellular localization of ARTD10 and observed that this protein shuttles between the nuclear and cytoplasmic compartments controlled by a Crm1-dependent NES and a region within ARTD10 that mediates nuclear uptake but does not contain any recognizable classical nuclear localization sequence (NLS). Moreover ARTD10 forms cytoplasmic bodies that are highly dynamic and can be linked to the autophagy system by interaction with p62/SQSTM1.

## Results

### ARTD10 interacts with nuclear MYC

**Figure 1:**
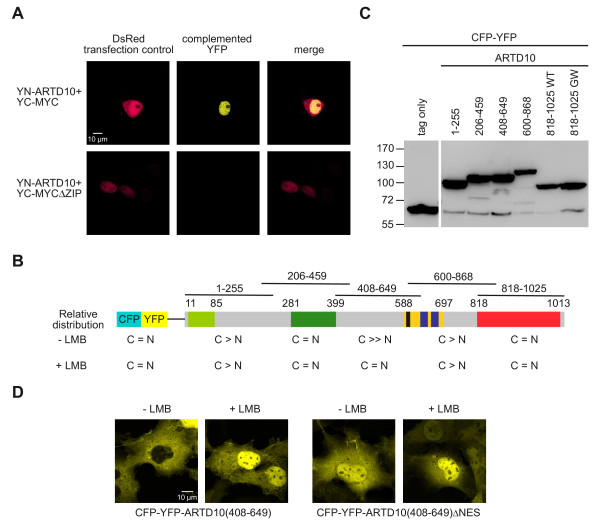
**Subcellular localization of ARTD10.A**. ARTD10 interacts with the oncoprotein MYC in the nucleus. The indicated fusion proteins were transiently expressed in HeLa cells. The expression of DsRed served as a transfection control. The appearance of yellow fluorescence was monitored by confocal microscopy. **B**. The scheme summarizes the domain structure of ARTD10 (light green, RNA recognition motif RRM; green, glycine-rich region; yellow, glutamate-rich region; black, nuclear export sequence; blue, ubiquitin-interaction motif; red, catalytic domain). The fusions of full-length ARTD10 and of overlapping fragments of ARTD10 to CFP-YFP are indicated. These fusion proteins were transiently expressed in COS7 cells and their subcellular distribution determined in the presence or absence of Leptomycin B (LMB). The relative distribution between the cytoplasmic and the nuclear compartment is indicated for each fusion protein (for representative micrographs see Figure 2B). **C**. The fusion proteins indicated in panel B were expressed transiently in COS7 cells and analyzed by Western Blotting using an anti-GFP antibody. **D**. The subcellular localization of CFP-YFP-ARTD10(408–649) and CFP-YFP-ARTD10(408–649)ΔNES was analyzed in transiently transfected COS7 cells in the presence or absence of LMB using confocal microscopy.

ARTD10 was identified as a direct interaction partner of the nuclear oncoprotein MYC [1]. This suggested that ARTD10 is localized at least in part in the cell nucleus. However our initial studies indicated that ARTD10 is mainly cytoplasmic [1]. Therefore we analyzed the ARTD10-MYC interaction in living cells by bimolecular fluorescence complementation (BiFC) [15]. Using a split-YFP system with N-terminal and C-terminal halves of YFP fused to ARTD10 and MYC, respectively, we detected a complemented YFP signal exclusively in the nucleus indicating that YN-ARTD10 and YC-MYC interact in this compartment (Figure 1A). Importantly, a YC-MYCΔZIP lacking the C-terminal leucine-zipper required for the direct interaction with ARTD10 did not reconstitute the YFP signal (Figure 1A). Therefore, we concluded that, although most ARTD10 is cytoplasmic in proliferating cells, ARTD10 interacts with MYC in the nucleus.

ARTD10 is exported from the nucleus by a Crm1-dependent NES [1]. Together with the experiment described above (Figure 1A), our findings suggested that ARTD10 shuttles between the nuclear and cytoplasmic compartments. Due to its relative molecular mass of ≈110,000 it is highly unlikely that ARTD10 is passing the nuclear envelope by simple diffusion. Rather the nuclear uptake is likely to be mediated by specific sequences or domains within ARTD10 such as an NLS [16]. However in silico analyses did not reveal any obvious NLS.

**Figure 2:**
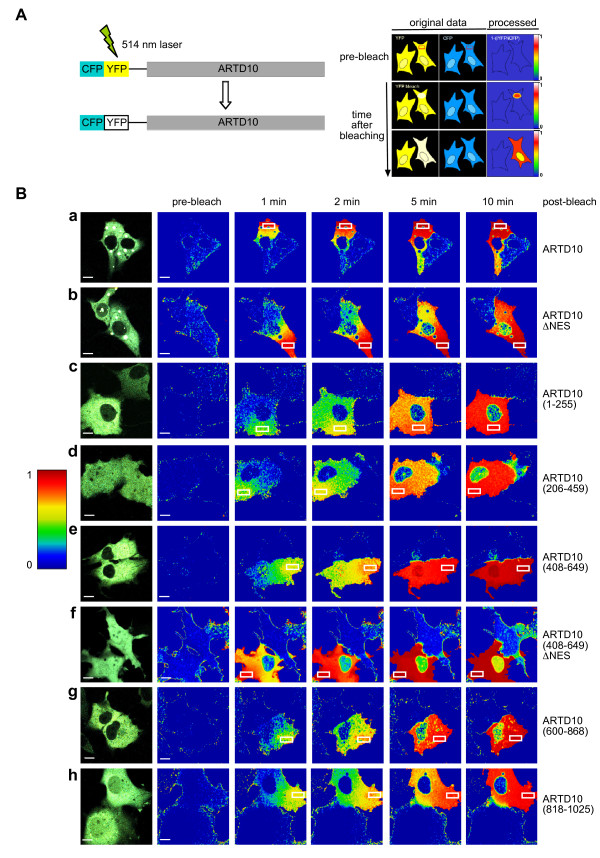
**ARTD10 shuttles between the cytoplasmic and nuclear compartments.A**. Schematic of the iFLAP imaging approach. Full length ARTD10 or different fragments of ARTD10 were fused N-terminally to CFP-YFP. The proteins were transiently expressed in COS7 cells. The YFP and CFP channels of the confocal microscope were adjusted so that the YFP and CFP fluorescence of the CFP-YFP-ARTD10 constructs appeared with almost identical signal intensities (iYFP ≅ iCFP). For each pixel of the image, using the formula intensity = 1–(iYFP/iCFP), a new image was calculated with almost zero signal intensity depicted in blue. Selective bleaching of YFP using the 514 nm laser of the confocal microscope within the rectangular bleaching-ROI (ROI = region of interest) generates a subpopulation of CFP-YFP-ARTD10 molecules that are only detected in the CFP-channel. These molecules generate a signal in the processed data channel. According to their intensities these signals are depicted in rainbow colors with blue representing weak and red strong signals. They diffuse or are transported through the cytoplasm and, depending on the protein analyzed, appear within the nucleus. A non-bleached cell is always included to demonstrate that signals are not generated as a result of bleaching upon image acquisition. **B**. The indicated proteins were expressed transiently in COS7 cells. A steady-state distribution of the different CFP-YFP-fusion proteins is shown in the panel on the left. The subcellular distribution of bleached fusion proteins is shown at different time points after bleaching. For control identical cells are shown prior to bleaching (pre-bleach). The scale bar indicates 10 μm.

To better understand ARTD10’s import/export mechanisms, we created fusion proteins of full-length ARTD10 and of overlapping ARTD10 fragments with an N-terminal fluorescent double tag consisting of CFP and YFP (Figure 1B). The size of at least 80 kDa of the resulting fusion proteins should largely prevent their diffusion into the nuclear compartment, thus allowing for the identification of regions within ARTD10 mediating nuclear uptake. Transient transfection of COS7 cells resulted in expression of the fusion proteins with no or very little cleavage of the CFP-YFP tag (Figure 1C). The steady-state subcellular localization of these fusion proteins was analyzed by confocal microscopy in the absence or presence of leptomycin B (LMB), an inhibitor of Crm1-dependent nuclear export (summarized in Figure 1B, see also Figure 2B). While three out of five fusion proteins were preferentially cytoplasmic, two fusion proteins were distributed equally between cytoplasm and nucleus (Figures 1B and 2B). Only CFP-YFP-ARTD10 (408–649), which contains the NES, showed an altered localization upon LMB treatment (Figure 1D). This fusion protein is exclusively cytoplasmic in untreated cells, but is predominantly nuclear upon LMB treatment. In line with this observation, the functional deletion of the NES by mutating key residues mirrored the effect of LMB (Figure 1D).

### A central region within ARTD10 confers rapid nucleo-cytoplasmic shuttling

The analysis of the steady-state localization of CFP-YFP fusion proteins provides only static information. To obtain dynamic information, we employed iFLAP (intramolecular fluorescence localization after photobleaching) [17,18]. This approach uses the CFP-YFP double tag and the selective bleaching of the YFP moiety in the cytoplasm, while the unbleached CFP serves as an intramolecular control. Because CFP and YFP are part of the same fusion protein, they are expressed in equimolar quantities and the ratio of both fluorophores can be used to visualize the movement of bleached fusion proteins within a cell (Figure 2A).

First we measured the redistribution of CFP-YFP-ARTD10, which localized exclusively to the cytoplasm when the steady-state distribution of the fusion protein was analyzed. Within the time frame of the experiment, little nuclear signal could be measured (Figure 2Ba). This might be the result of slow nuclear import or rapid export of ARTD10. Mutating the NES in CFP-YFP-ARTD10ΔNES was sufficient to substantially enhance the nuclear signal (Figure 2Bb). This suggested that due to the reduced export rate, accumulation of ARTD10 was now detectable in the nucleus. Thus ARTD10 translocated into the nucleus where it can be visualized when bound to a nuclear protein such as MYC (Figure 1A) or when the NES-dependent export is inhibited (Figure 2Bb) [1].

To define further the regions in ARTD10 relevant for nucleo-cytoplasmic transport, we measured the redistribution of different fusion proteins containing overlapping portions of ARTD10 (Figure 1B). Most of the fusion proteins redistributed rapidly within the cytoplasm, while little or no exchange with the nuclear pools occurred (Figure 2B). However, the exchange of CFP-YFP-ARTD10(408–649) between the cytoplasm and the nucleus occurred as fast as the diffusion within the cytoplasm, indicating a rapid nucleo-cytoplasmic shuttling of this fusion protein (Figure 2Be). Inactivating the NES reduced the velocity of the nucleo-cytoplasmic shuttling in CFP-YFP-ARTD10 (408–649)ΔNES, but enhanced considerably the nuclear steady-state levels (Figure 2Bf). One possible explanation for this finding is the saturation of the nuclear compartment as a result of the reduced export, which may inhibit further uptake of fusion proteins. This indicates that the NES between amino acids 598 and 607, which mediates rapid export, is important for rapid shuttling. Moreover, the experiments described above suggested that ARTD10 (408–649) contains a sequence that promotes nuclear import. Two additional regions have weak NLS function. One is in ARTD10(600–868), a fragment that did not accumulate in the nucleus but the iFLAP analysis showed a slow exchange of protein between the cytoplasm and the nucleus (Figure 2Bg). A different behavior was observed with the catalytic domain, ARTD10(818–1025). This fragment showed nuclear localization under steady-state conditions, but within the time frame of the iFLAP experiment no nuclear transport was visible (Figure 2Bh), suggesting that it very slowly accumulates in the nucleus and remains there. Thus besides the more prominent region promoting nuclear uptake contained in ARTD10 (408–649), other regions of ARTD10 might contribute to the observed shuttling of the protein between cytoplasmic and nuclear compartments.

### A conserved region within ARTD10 constitutes a non-conventional NLS

**Figure 3:**
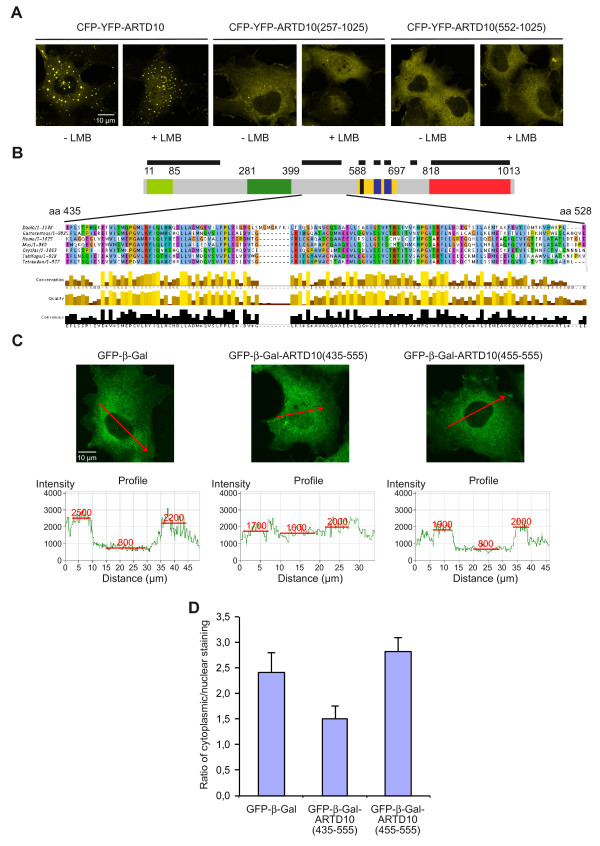
**A conserved ARTD10 region encompassing amino acids 435–528 mediates nuclear uptake.A**. The indicated CFP-YFP fusion proteins were expressed transiently in COS7 cells in the presence or absence of LMB. Their subcellular distribution was determined by confocal microscopy. **B**. Above the schematic structural organization of ARTD10, evolutionary conserved regions of the protein are denoted with a black bar. The conserved region in the central part of ARTD10 encompassing amino acids 435–528 is shown for zebra fish, stickleback, human, mouse, medaka, and two pufferfish (from top to bottom). **C**. GFP-β-Gal, GFP-β-Gal-ARTD10(435–555), and GFP-β-Gal-ARTD10(455–555) were expressed transiently in COS7 cells and the subcellular distribution of the different fusion proteins analyzed by confocal microscopy. Below the micrographs intensity profiles are shown, which were measured from the areas indicated by the red arrow. Mean intensities were determined in the cytoplasm and in the nucleus as indicted in the profiles. These intensities were used to assess the ratio of cytoplasmic/nuclear staining as summarized in panel D. **D**. Summary of the ratio of mean cytoplasmic vs. mean nuclear florescence of the indicated fusion proteins (examples of single cells are shown in panel C).

In order to test the hypothesis that ARTD10 (408–649) contains an NLS function, we created N-terminal deletion mutants of ARTD10 and compared the subcellular localization of CFP-YFP fusion proteins in the absence or presence of LMB. Full-length ARTD10 entered the nucleus upon LMB treatment. The deletion of the N-terminal 256 amino acids harboring an RNA recognition motif (RRM) did not affect the nuclear import of ARTD10 (257–1025) in the presence of LMB. Importantly, the deletion of the N-terminal half of ARTD10, i.e. amino acids 1–551, abrogated the ability of ARTD10 to enter the nuclear compartment (Figure 3A). Along with the findings from the previous localization experiments, this indicated that the region encompassing amino acids 408–551 might be relevant for nuclear import. This part of the protein, as it is also true for full-length ARTD10, does not reveal a classical NLS using bioinformatic prediction methods (data not shown). Because a region with NLS function is most likely relevant for the physiological function of ARTD10, we expected it to be conserved. Therefore we analyzed the cross-species conservation of this region of ARTD10 by a T-Coffee alignment. This revealed a strong conservation of a thus far uncharacterized region in the central part of ARTD10 spanning amino acids 435–528 (Figure 3B). We hypothesized that this region might be relevant for nuclear import. This was tested by fusing amino acids 435–555 of ARTD10 to GFP-β-Gal, a well-established model protein to test NLS functions [19]. While GFP-β-Gal was cytoplasmic, the GFP-β-Gal-ARTD10 (435–555) fusion protein was partially nuclear (Figure 3C), indicating that this conserved region was sufficient to drive nuclear import. Importantly, the deletion of the N-terminal 20 amino acids of the conserved region was sufficient to block nuclear uptake of the fusion protein (Figure 3C). We have estimated the mean fluorescent signal in the cytoplasm and the nucleus for these proteins and determined the ratio of the signals (Figure 3D). This further substantiates the conclusion that the integrity of the ARTD10 (435–555) region is important for nuclear localization. Together these experiments define a central, conserved region in ARTD10 that possesses NLS function.

### The ratio of cytoplasmic and nuclear ARTD10 is tightly controlled

**Figure 4:**
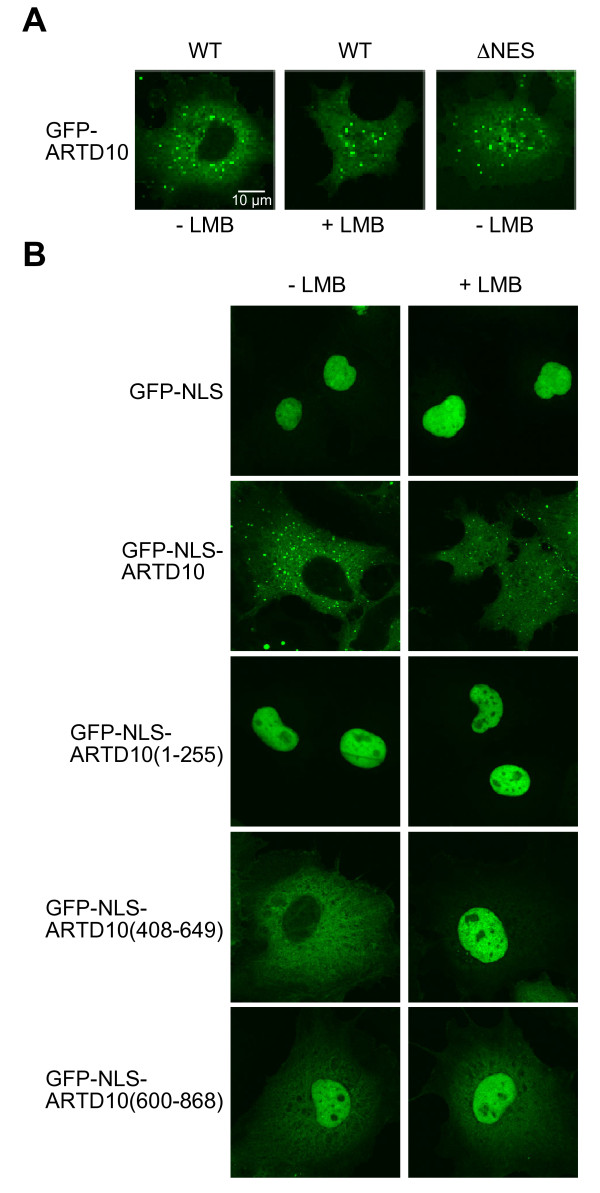
**The ARTD10 region spanning amino acids 600–868 contain additional element(s) that control subcellular localization.A**. GFP-ARTD10 and GFP-ARTD10ΔNES were expressed transiently in COS7 cells. The cells were treated with or without LMB for 2 h. The subcellular distribution of the fusion proteins was measured by confocal microscopy. **B**. Identical experimental set-up for the indicated fusion proteins as in panel A. NLS, the nuclear localization sequence of the large T antigen of simian virus 40.

Although the identified unconventional NLS enabled GFP-β-Gal to enter the nucleus, it was surprising that GFP-β-Gal-ARTD10 (435–555) did not enrich in the nucleus but only distributed equally between cytoplasm and nucleus (Figure 3C). Because classical NLS elements are able to drive efficient nuclear accumulation of GFP-β-Gal [19], we decided to further investigate the interplay between the import and export mechanisms. We used a set of GFP fusion proteins with GFP-ARTD10 behaving identically to CFP-YFP-ARTD10. In the presence of LMB, GFP-ARTD10 distributed equally between cytoplasmic and nuclear compartments, similar to the finding with GFP-ARTD10ΔNES (Figure 4A). First we asked whether the classical NLS of the large T-antigen of Simian Virus 40 (SV40) was able to support nuclear localization of ARTD10. While GFP-NLS(SV40) was exclusively nuclear, GFP-NLS (SV40)-ARTD10 was still cytoplasmic, denoting that the NES of ARTD10 counteracted efficiently the SV40-NLS (Figure 4B). In the presence of LMB, GFP-NLS(SV40)-ARTD10 distributed equally. This result argues for either an additional, Crm1-independent export mechanism or for sequences that suffice to retain at least part of the protein in the cytoplasm, despite the strong NLS of SV40. Also there might be mechanisms that limit the amount of ARTD10 in the nucleus. Together additional elements and motifs might contribute to control the ratio of cytoplasmic and nuclear ARTD10.

In order to localize a potential second export signal or a retention signal, we created fusion proteins of GFP-NLS and overlapping ARTD10 fragments analogous to the ones described in Figure 1B. When we analyzed the localization of these fusion proteins, most of them located exclusively to the nucleus as expected from the presence of the SV40-NLS, which is exemplary shown for GFP-NLS(SV40)-ARTD10 (1–255) (Figure 4B). ARTD10 (408–649) harboring the Crm1-dependent NES and fused to GFP-NLS(SV40) localized mainly in the cytoplasm as seen with full-length ARTD10. This indicated that ARTD10’s NES was indeed able to outpace the SV40-NLS-driven import. In contrast to the localization of GFP-NLS (SV40)-ARTD10, GFP-NLS (SV40)-ARTD10 (408–649) accumulated efficiently in the nucleus upon LMB treatment. For GFP-NLS (SV40)-ARTD10 (600–868) we found that it displayed reduced nuclear import in comparison to GFP-NLS (SV40), both in the presence or absence of LMB (Figure 4B). Thus, we concluded that a so far uncharacterized, but transferable motif is located within amino acids 600–868 representing either a potential Crm1-independent export signal or a cytoplasmic retention signal. In summary, the subcellular localization of ARTD10 is regulated by a complex interplay of a non-conventional NLS, a classical Crm1-dependent NES and at least one further signal counteracting the nuclear import of ARTD10.

### ARTD10 bodies are highly dynamic structures

**Figure 5:**
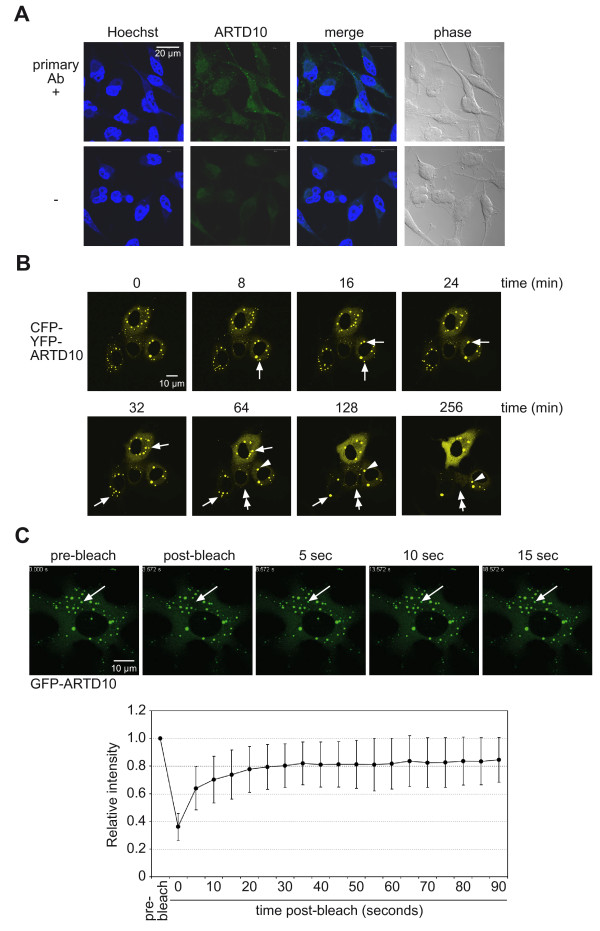
**ARTD10 forms dynamic bodies in cells.A**. HeLa cells were fixed and stained with an ARTD10-specific antibody (E09). The DNA was labeled with Hoechst 33258. **B**. CFP-YFP-ARTD10 was expressed in COS7 cells. A frame with 4 cells was analyzed using live-cell imaging for 256 minutes with one picture taken every 4 minutes. Arrows indicate ARTD10 bodies that fuse, the arrowheads a body that moves within the cell, and double-arrows bodies that appear and disappear. **C**. A single ARTD10 body was bleached at high laser intensity (upper panel). The recovery of the fluorescence signal was monitored by live-cell imaging. Using the mean of 12 bleaching experiments in different cells the FRAP kinetics were quantified and are displayed with standard deviations (lower panel).

The expression of CFP-YFP-ARTD10 or GFP-ARTD10 resulted in the formation of characteristic bodies, which formed mainly in the cytoplasm but occurred also in the nucleus, particularly upon LMB treatment (e.g. Figures 3A and 4A). These bodies were also detectable when endogenous ARTD10 was analyzed in HeLa and U2OS cells (Figure 5A and data not shown). To determine whether these ARTD10 bodies are dynamic, their mobility was analyzed by live-cell imaging of CFP-YFP-ARTD10 transiently expressed in COS7 cells (Figure 5B). We observed that ARTD10 bodies represent dynamic structures, which tended to fuse over time (arrows in Figure 5B), moved considerable distance (arrowheads in Figure 5B), and formed de novo and also disappeared again (double-head arrows in Figure 5B). Thus these findings demonstrate that ARTD10 bodies are mobile in cells.

To address whether ARTD10 molecules within single bodies are dynamic, we subjected GFP-ARTD10 to FRAP (fluorescence recovery after photobleaching) assays. ARTD10 bodies were bleached individually at high laser intensity and the fluorescence recovery was monitored by live-cell imaging. We observed rapid recovery of the fluorescence within ARTD10 bodies indicating a rapid exchange of GFP-ARTD10 molecules between ARTD10 bodies and cytoplasmic GFP-ARTD10 and/or molecules from other bodies (Figure 5C). Our results indicate that ARTD10 bodies do not represent static, immobile structures of deposited protein, but instead are highly dynamic.

### ARTD10 bodies are recognized by the autophagy adaptor protein p62

**Figure 6:**
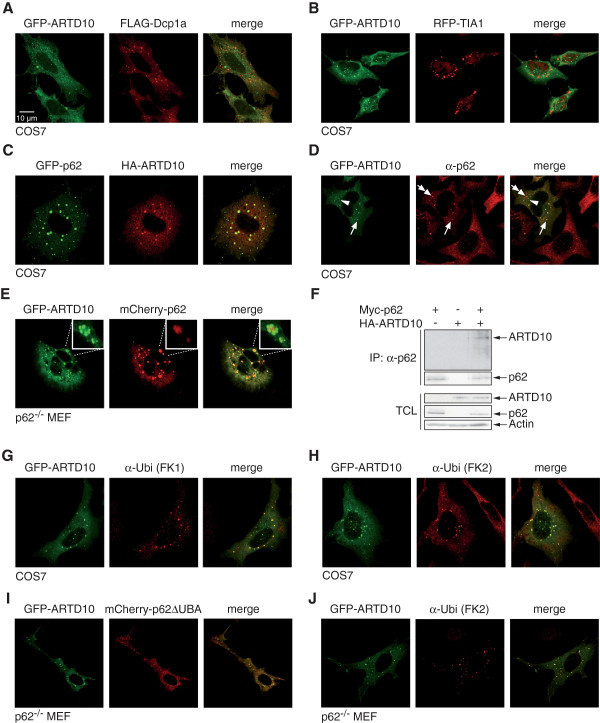
**ARTD10 colocalizes with p62 bodies.A**. GFP-ARTD10 and FLAG-Dcp1a, a P-body component, were co-expressed in COS7 cells. FLAG-Dcp1a was stained with a FLAG-specific antibody and co-localization of the two proteins was evaluated by confocal microscopy. **B**. GFP-ARTD10 and RFP-TIA1, a marker for stress granules, were co-expressed in COS7 cells and the localization evaluated. **C**. HA-ARTD10 and GFP-p62 were co-expressed in COS7 cells. HA-ARTD10 was stained with a HA-specific antibody and co-localization of the two proteins was evaluated. **D**. GFP-ARTD10 was expressed in COS7 cells. Endogenous p62 was stained with specific antibodies. Arrows indicate bodies that contain ARTD10 and are positive for p62. Double-arrows and arrowheads indicate p62 and ARTD10 only positive bodies, respectively. **E**. GFP-ARTD10 and mCherry-p62 were expressed in p62^−/−^ MEF and their subcellular localization evaluated. **F**. HA-ARTD10 and Myc-p62 were co-expressed in HEK293 cells. p62 and associated proteins were immunoprecipitated and analyzed by Western blotting. For control the expression of HA-ARTD10, Myc-p62, and actin was determined as indicated. **G**. COS7 cells expressing GFP-ARTD10 were stained for ubiquitin using mAb FK1, which recognizes ubiquitin polymers. **H**. As in panel F but with mAb FK2, which recognizes both ubiquitin monomers and polymers. I. GFP-ARTD10 and mCherry fusion protein with a p62 mutant unable to interact with ubiquitin (mCherry-p62ΔUBA) were expressed in p62^−/−^ MEFs. The localization of the proteins was determined. I. GFP-ARTD10 was expressed in p62^−/−^ MEFs and the cells were stained for ubiquitin using FK2.

Different types of cytoplasmic bodies have been described, including RNA-associated structures like P-bodies and stress granules [20]. Because ARTD10 possesses an N-terminal RRM, we tested whether ARTD10 bodies co-localize with components of P-bodies or stress granules. Therefore, we co-expressed GFP-ARTD10 and FLAG-Dcp1a, a P-body component, or the stress granule marker RFP-TIA1. Both markers localized to discrete cytoplasmic bodies, but no co-localization with ARTD10 bodies could be detected indicating that these structures are distinct from P-bodies or stress granules (Figure 6A and B). In addition, we tested for co-localization between ARTD10 and several other described cytoplasmic substructures including mitochondria, Golgi, endoplasmic reticulum (ER), and endosomes. No co-localization could be detected in any of these co-stainings (data not shown).

Another structure that forms dynamic cytoplasmic bodies are autophagosomes [21]. Therefore we analyzed the co-localization of ARTD10 and p62. The latter is an autophagy adaptor protein that binds to LC3-II and to ubiquitin and forms cytoplasmic foci [22]. Indeed, when GFP-p62 and HA-ARTD10 were co-expressed, we detected a high degree of co-localization (Figure 6C). GFP-ARTD10 co-localized also with endogenous p62, although not all ARTD10 bodies were p62 positive and vice versa (Figure 6D). The analysis of these stainings revealed that the ARTD10 and the p62 bodies did not completely overlap but seemed to represent two bodies in close proximity. To further evaluate this finding, we co-expressed low amounts of GFP-ARTD10 and mCherry-p62 in p62^−/−^ mouse embryonic fibroblasts (MEF). The analysis of the bodies in this experimental set-up demonstrated that the p62 bodies were decorated with ARTD10 bodies (Figure 6E). Thus these findings suggest that the p62 bodies serve as docking sites for ARTD10 bodies. In support overexpressed ARTD10 and p62 could be co-immunoprecipitated (Figure 6F), although the interaction appeared rather weak.

p62 recognizes ubiquitinated cargo destined to autophagosomes via its ubiquitin-associated (UBA) domain, a ubiquitin-binding module [22]. Therefore we addressed whether ARTD10 bodies are associated with ubiquitin-protein conjugates. Cells expressing GFP-ARTD10 were stained with two different monoclonal antibodies that recognize ubiquitinated proteins, FK1 and FK2. Whereas FK2 recognizes mono- and poly-ubiquitinated proteins, FK1 is restricted to the recognition of poly-ubiquitinated proteins [23]. Both antibodies stained GFP-ARTD10 bodies indicating that these contain ubiquitin and/or poly-ubiquitin (pUb) (Figure 6G and H). Thus one possibility is that p62 interacts with ARTD10 bodies through ubiquitin. Indeed a p62 mutant that is unable to bind to ubiquitin (mCherry-p62ΔUBA) no longer co-localized with ARTD10 (Figure 6I). In cells lacking p62, ARTD10 bodies were still ubiquitin positive (Figure 6J). It is possible that other ubiquitinated proteins are part of ARTD10 bodies or that ARTD10 itself is ubiquitinated. In support of the latter, in preliminary experiments ARTD10 was purified with overexpressed His_6_-Ub (data not shown), indicating that ARTD10 itself is ubiquitinated.

## Discussion

**Figure 7:**
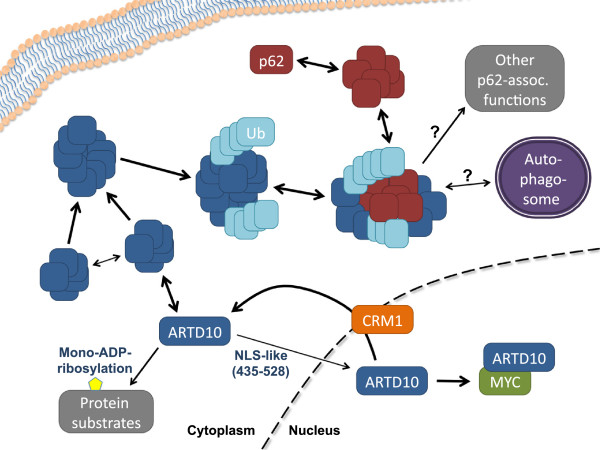
**Model summarizing the trafficking and the molecular interaction of ARTD10.** ARTD10 forms cytosolic bodies that are highly dynamic, in regard to both the intracellular mobility and the exchange of subunits. Moreover ARTD10 translocates into the nucleus dependent on an NLS-like domain and is rapidly exported in a CRM1 and NES controlled process. In the nucleus ARTD10 interacts with the oncoprotein MYC. The cytosolic ARTD10 bodies are ubiquitinated and interact with p62, a ubiquitin receptor associated with autophagy. The functional relevance of these bodies may be in autophagosomal processes and/or in other as yet undefined p62-dependent functions.

Our findings document that ARTD10, the first identified intracellular mono-ADP-ribosyltransferase, shuttles between the nuclear and cytoplasmic compartments (Figure 7). Under steady-state conditions the majority of ARTD10 molecules reside in the cytoplasm, predominantly the consequence of a highly efficient NES (L_598_LATLEGLDL) that represents a classical Leu-rich, Crm1-dependent element [24]. An additional region that contributes to the cytoplasmic localization is confined to amino acids 600–868 that may function as Crm1-independent export signal or a cytoplasmic retention signal. Moreover ARTD10(600–868) also allows some nuclear uptake, indicating that this region may contribute to shuttling independent of the more prominent NES and the amino acid 435–528 region. Although the uptake of ARTD10 into the nucleus seems rather slow, as measured by iFLAP, and difficult to detect due to the highly efficient NES, we were able to identify a sequence that mediates nuclear localization. This fragment encompassing amino acids 435–528 represents an evolutionary highly conserved sequence (Figure 3). Proteins that are transported into the nucleus interact with members of the karyopherin-βs (Kapβs, also known as importins and exportins), either directly or in complex with adaptor proteins such us Kapαs [25]. However no recognizable NLS can be detected in ARTD10(435–528). In particular no basic clusters are present within these 94 amino acids that could form a classical NLS, either a monopartite or bipartite element [25]. Also no K/R/H-PY element exists that has been postulated to represent a recognition motif for Kapβ2 [26]. To address whether ARTD10(435–528) can interact with karyopherins, we have begun to test for direct interaction. So far we have analyzed Kapα1-7, but were unable to detect binding (our unpublished findings). Thus it remains open how the ARTD10(435–528) fragment mediates nuclear localization.

Because we observed that ARTD10 interacts with MYC in the nucleus (Figure 1), we considered that MYC, which contains at least two NLS [27], might contribute to the nuclear uptake of ARTD10. This was also indicated by the observation that ARTD10(600–868) and ARTD10(818–1025) showed some nuclear localization when measured by iFLAP or under steady-state conditions, respectively (Figure 2), and that the C-terminal one-third of ARTD10 can interact directly with MYC [1]. However co-expressing MYC had no measurable impact on the subcellular distribution of ARTD10, neither in the presence nor in the absence of the NES (our unpublished findings).

Although the amino acid 435–528 fragment is evolutionary conserved in ARTD10 (Figure 3), it does not score in a search for known protein domains. We noticed RXL motifs that can mediate the interaction with cyclins. Indeed we have noticed that ARTD10 is a substrate for cyclin-dependent kinases but the relevance of the respective phosphorylation sites for the subcellular localization of ARTD10 has not been clarified yet. Together it is possible that ARTD10(435–528) interacts with a karyopherin but it would appear that this interaction does not follow any of the known rules. Furthermore we cannot exclude that this region of ARTD10 connects to a protein that possesses an NLS and thus serves as a piggyback transport mechanism for ARTD10 into the nucleus.

ARTD10 forms prominent discrete bodies in the cytoplasm. These structures do not seem to be artifacts of overexpressed proteins because staining of endogenous ARTD10 reveals similar bodies (Figure 5). Moreover they are highly dynamic and the subunits exchange quickly between the bodies and the cytoplasmic environment, arguing against non-specific protein aggregates. The analysis of different ARTD10 mutants showed that the full-length protein is required for efficient body formation. In particular different fragments did not form bodies (Figure 2). This was also the case for ARTD10(1–255), which contains the RRM. Moreover deletion of the N-terminal 256 amino acids in ARTD10 (257–1025) considerably reduced body formation (Figure 3). Therefore it seems that the fragment with the RRM is important but not sufficient for body formation. RRMs are among the most abundant protein domains identified in eukaryotes [28]. RRMs were originally described as domains that mediate interaction with RNA, but RRMs have also been found to mediate protein-protein interaction [29]. Because of the involvement of proteins with RRM domains in many aspects of RNA processing and translation, we tested whether ARTD10 co-localized with P-bodies or stress granules, both structures that are associated with RNA storage and processing [30,31]. However we were unable to detect any interaction of ARTD10 with markers that define either P-bodies or stress granules (Figure 6 and data not shown). We also applied different forms of stress, including ROS, which have been demonstrated to stimulate the formation of stress granules. Again these treatments were not sufficient to promote ARTD10 co-localization with either stress granules or P-bodies (data not shown). Thus despite the presence of an RRM, ARTD10 seems not to be involved in RNA processing associated with these structures. Whether ARTD10 participates in another aspect of RNA processing remains undefined; for example the ability of ARTD10 to shuttle between the cytoplasmic and nuclear compartments may suggest a role in RNA transport.

Furthermore ARTD10 did not co-localize with a number of distinct markers that included GM130 (for Golgi), CFP-ER (for ER), Rab5 and EEA1 (for endosomes). The only protein we were able to identify that co-localized was p62/SQSTM1. This protein has multiple functions, one of its best-studied is as an adaptor in autophagy [22]. p62 interacts with LC3-II, the phosphatidylethanolamine-bound form of ATG8, which is located on the phagophore. p62 interacts with poly-ubiquitinated proteins through its UBA domain, carrying either K63- or K48-linked pUb, thereby targeting poly-ubiquitinated substrates to the autophagosome [32]. This process is of considerable importance both in normal and in pathophysiological conditions. For example protein aggregates or damaged mitochondria are removed from cells by this mechanism and failure to do so may result in diseases such as cancer or neurodegeneration [22,33]. Although the co-localization of ARTD10 with p62 was readily visible in our experiments, the interaction was weak as judged from the co-immunoprecipitation experiments that only detected overexpressed proteins (Figure 6). The co-localization required the UBA domain, which recognizes pUb. A possible consequence of the interaction of ARTD10 with p62 is that ARTD10 or ARTD10 bodies are targeted to autophagosomes. Whether ARTD10 is a substrate of the autophagosomal machinery, whether it is involved in targeting cargo to autophagosomes, or whether it functions as a regulator, through e.g. mono-ADP-ribosylation, of the autophagosomal process remains to be determined.

## Conclusions

We have identified regions in ARTD10 that control its subcellular localization dynamically (Figure 7). Most prominent is a nuclear export sequence that efficiently shuttles ARTD10 into the cytosol in a Crm1-dependent mechanism. A domain of almost 100 amino acids is primarily responsible for the nuclear uptake, but does not contain any classical nuclear localization sequence. The ARTD10 – MYC complex is exclusively nuclear, but MYC itself is unable to drive ARTD10 into the nucleus. Moreover ARTD10 forms bodies that are dynamic both in their location and in their composition. These bodies associate at least in part with the poly-ubiquitin receptor p62 dependent on ARTD10 being ubiquitinated. Thus our findings suggest an interaction of ARTD10 with autophagosomal processes, possibly as a regulatory factor.

## Methods

### Cell culture, treatments and transfections

COS7 (African green monkey kidney), HEK293 (human embryonic kidney), and HeLa (human cervical carcinoma) cells were grown at 37°C, 5% CO2 in Dulbecco’s Modified Eagle’s Medium (DMEM) with GlutaMAX (Invitrogen, Karlsruhe, Germany) supplemented with 10% fetal calf serum. Leptomycin B (Calbiochem, Darmstadt, Germany) treatment was performed at 20 nM typically for 4 h. Cells were transfected with FuGene HD (Roche, Mannheim, Germany) according to the manufacturer’s instructions.

### Plasmids and site-directed mutagenesis

Bimolecular fluorescence complementation (BiFC) was performed using the plasmids pBiFC-MycYC155 or pBiFC-MYCΔZIPYC155 essentially as described [34]. The cDNA for ARTD10 was cloned into pBiFC-YN155. 2.25 μg of the indicated plasmids were transiently transfected into HeLa cells together with 0.25 μg of the transfection control plasmid pDsRed-Monomer-N1 (Clontech Laboratories, Mountain View, CA, USA) using ExGen500 (Fermentas, St. Leon-Rot, Germany) according to the manufacturers recommendations. After 2 days the cells were grown for 1.5 hours at 30°C for maturation and fluorescence was assessed by confocal microscopy. pECYFP has been described before [35]. The plasmids encoding p62 were described previously [36,37]. The plasmids containing deletion mutants of ARTD10 were obtained using the Gateway system (Invitrogen) according to the manufacturer’s instructions. For this purpose the desired regions were amplified by PCR on pEVRF0-HA-ARTD10 [1] using Gateway-compatible PCR primers and introduced into the pDONR/Zeo entry vector (Invitrogen) by a Gateway BP reaction. All PCR reactions were performed with the Phusion polymerase (NEB, Frankfurt, Germany) according to the manufacturer’s instructions.

The vectors pECYFP and pEGFP were made compatible to the Gateway system (Invitrogen) by insertion of the Gateway cassette reading frame B into the *Sma*I site of the vectors. The resulting destination vectors GW-pECYFP and GW-pEGFP were used in a Gateway LR reaction along with suitable entry vectors to create expression vectors for CFP-YFP-tagged or GFP-tagged proteins, respectively. The destination vector GW-pEGFP-NLS was generated by digestion of GW-pEGFP with *Hind*III and dephosphorylation with FastAP (Fermentas). Subsequently, the hybridized oligonucleotides NLS-for (AGCTCCAAAGAAGAAGCGAAAGGTA) and NLS-rev (AGCTTACCTTTCGCTTCTTCTTTGG) encoding the nuclear localization sequence of SV40 large T antigen were ligated into the restricted vector. In the case of GW-pEGFP-β-Gal the restricted GW-pEGFP was ligated with a PCR-product containing the open reading frame of β-Gal. The latter was obtained by PCR on a CMV-β-Gal plasmid using the primers β-Gal-for (GTCTAAGCTTCGGTCGTTTTACAACGTC GTGACTGG) and β-Gal-rev (GTCTCGAAGCTTTTTTTGACACCAGACCAAC TGGTAATG) and subsequently digested with *Hind*III to generate compatible ends. Plasmids encoding full-length ARTD10 or point mutations thereof were constructed by classical cloning procedures. In the case of pEGFP-based constructs, pEGFP as well as pEVRF0-HA-ARTD10 plasmids were sequentially digested with *Xba*I and *Kpn*I and subsequently ligated using T4 ligase (Fermentas).

The directed introduction of point mutations into ARTD10-encoding plasmids was achieved by using the QuickChange Site-directed mutagenesis kit (Stratagene, La Jolla, CA, USA) according to the manufacturer’s instructions.

### Antibodies

ARTD10 was detected using rat monoclonal antibodies 5H11 (IgG1 subclass that recognizes amino acids 300–350), rabbit polyclonal sera 890–6 [1], and purified rabbit antibodies (E09) generated against ARTD10(206–459) by Eurogentec (Liege Science Park, Seraing, Belgium).

The following primary antibodies were used in immunofluorescence experiments: α-FLAG (clone M2, 1/1000; Sigma, Taufkirchen, Germany), α-HA (clone 16B12, 1/1000; Covance, Munich, Germany), α-p62 (clone 3, 1/200; BD Biosciences, Heidelberg, Germany)), α-Ubiquitin (FK1, 1/500; Biomol, Hamburg, Germany), α-Ubiquitin (FK2, 1/500; Biomol). The following secondary antibodies were used in immunofluorescence experiments: goat α-mouse-IgG-Alexa555 (1/1000; Invitrogen), goat α-mouse-IgM-Alexa555 (1/1000; Invitrogen).

The following primary antibodies were used for immunoblots: α-GFP (clone 9F9.F9, 1/10000; Rockland Immunochemicals, Gilbertsville, PA, USA), α-HA (clone 3F10, 1/1000; Roche). The following secondary antibodies were used for immunoblots: goat α-mouse-HRP (1/5000; Dianova, Hamburg, Germany), goat α-rat-HRP (1/5000; Dianova).

### Immunofluorescence

24 hours post-transfection, cells were washed twice with PBS and fixed for 15 minutes in 3.7% paraformaldehyde/PBS at ambient temperature. The cells were permeabilized for 10 minutes with 0.1% Triton-X-100/PBS or, if vesicular structures were stained, with 40 μg/ml Digitonin (Sigma) in PBS. Non-specific binding was blocked using 3% goat serum (Invitrogen)/PBS for 30 minutes at ambient temperature. Antibodies were diluted in 1% goat serum/PBS. Primary antibodies were incubated for 45 minutes in a humid chamber at 37°C. After three washes with PBS secondary antibodies were incubated for 45 minutes in a humid, dark chamber at 37°C. Cells were washed three times with PBS, washed once with ddH_2_O, incubated with 200 ng/ml Hoechst 33258 (Sigma) in ddH_2_O for two minutes and mounted onto glass slides with Mowiol 4–88 (Calbiochem). Confocal images were taken with a Zeiss LSM 510 (Carl Zeiss AG, Jena, Germany) using a water-corrected 63x objective lens NA 1.2.

### Time-lapse microscopy

COS-7 cells were grown on 42 mm glass slides and transiently transfected with pECYFP-PARP10. 24 hours post-transfection the coverslide was placed in a thermostat-controlled (37°C) and CO_2_-controlled (5%) perfusion chamber (Pecon, Erbach, Germany). Cells were imaged on a Zeiss LSM510 confocal microscope (Carl Zeiss AG, Jena, Germany) using a 63x objective lens NA 1.2 and Immersol (Carl Zeiss AG, Jena, Germany). Confocal images were taken using an autofocus function every four minutes for about eight hours.

### Inverted Fluorescence Localization After Photobleaching (iFLAP)

iFLAP analysis was performed as described previously [17].

### Fluorescence Recovery After Photobleaching (FRAP)

COS-7 cells were grown on 42 mm glass slides and transiently transfected with pEGFP-PARP10. 24 hours post-transfection the coverslide was placed in a thermostat-controlled (37°C) and CO_2_-controlled (5%) perfusion chamber (Pecon, Erbach, Germany). Confocal images were taken with a Zeiss LSM 510 (Carl Zeiss AG, Jena, Germany) using a water-corrected 63x objective lens NA 1.2. Highly fluorescent PARP10 bodies were bleached using the 488 nm emission line with 100% laser power and 200 iterations. Twenty pictures including a pre-bleach image were taken every five seconds. Fluorescence intensity at the bleached area was measured for every time point. For quantification, the measured intensities were normalized against the pre-bleaching value and the mean of twelve independent experiments was calculated for every single time point. The results of all experiments were plotted onto a graph displaying the normalized mean fluorescence intensities and the according standard deviations of the mean (SD).

### Immunoprecipitation

HEK293 cells were transfected transiently using the calcium phosphate coimmunoprecipitation method as described before [38]. The cells were treated with 0.5 mM dimethyl 3,3′-dithiobispropionimidate (Thermo Fisher Scientific, Bonn, Germany) in PBS at room temperature for 30 minutes. The cells were washed with 200 mM Tris–HCl/PBS and subsequently lysed in antibody buffer (20 mM Tris–HCl, pH 7.5; 50 mM NaCl; 0.5% NP-40; 0.5% DOC; 0.5% SDS; 0.5% Trasylol, 1 mM EDTA; 20 mM N-ethylmaleimide, Proteoblock 1:100). The cleared lysates were incubated with antibodies as specified.

## Abbreviations

ARTD: ADP-ribosyltransferase; AIF: Apoptosis-inducing factor; BiFC: Bimolecular fluorescence complementation; ER: Endoplasmatic reticulum; FRAP: Fluorescence recovery after photobleaching; iFLAP: Intramolecular fluorescence localization after photobleaching; Kapβ: Karyopherin-β; LMB: Leptomycin B; MEF: Mouse embryonic fibroblasts; NES: Nuclear export sequence; NLS: Nuclear localization sequence; pUb: Poly-ubiquitin; RRM: RNA recognition motif; SV40: Simian Virus 40; UBA: Ubiquitin-associated domain.

## Competing interests

The authors declare no competing financial interests.

## Authors’ contributions

HK and AH performed the majority of experiments, including all live-cell imaging; TL, AF PV and BLi studied the interaction of p62 with ARTD10; JL-F analyzed the interaction of MYC with ARTD10; KLHF, NH and EK prepared and tested the antibodies used; TJ, GM-N and BLü supervised the work and wrote the manuscript. All authors read and approved the final manuscript.

## Authors’ information

HK and AH were graduate students when they performed the experiments. HK is at Abbott GmbH since 2 years and AH is a post-doctoral fellow in the laboratory of Dr. Hua E. Yu at the Beckman Research Institute, City of Hope National Medical Center, Duarte, California since 3 years.
